# Malformation of the Genitals

**Published:** 1845-01

**Authors:** 


*Malformation of the Genitals.*—Mr. Terry examined a child, aged two months, at the Northampton General Infirmary, on account of a malformation in the organs of generation. The mother, and grandmother, who came with the child, said that the external opening was closed, and the water came the wrong way, and that she was born so. At first, it appeared to be a common case of imperforate vagina. There was, however, a little fulness, and a slight degree of prominence, at the lower part of the supposed closed orifice, and, on proceeding to separate the adhesion, which required no cutting, and very little force, there was found something which looked like a penis.

The parts were separated more extensively, and the parts which had been concealed more minutely examined; a glans penis and prepuce were then discernible, and the penis was complete, though confined and bound down to the neighbouring parts. The vagina was sought for, but in vain. The scrotum, with one testicle down, and the other descending, was gradually developed, and the little patient was presented as an entirely male child. There was a slight indentation at the orifice of the urethra, but the canal was impervious. and the urine passed through a little opening behind the corona glandis, just at the insertion of the frænum preputii. There was a large quantity of loose cellular substance, covered by integument in the neighbourhood of the parts, so that even after the separation of all the adhesions, the slightest lateral pressure with the fingers gave again the appearance of a closed vagina, covering and entirely hiding the penis and scrotum as before. The case is detailed in the *Provincial Medical Journal.*—*Ibid.*

